# Comparative study of coronary artery disease prediction: conventional QRISK3 versus enhanced machine learning models combined with particle swarm optimisation algorithm

**DOI:** 10.1136/openhrt-2025-003422

**Published:** 2025-10-31

**Authors:** Wigaviola Socha Purnamaasri Harmadha, Dennis Wang, Mohsin Masood

**Affiliations:** 1 National Heart and Lung Institute, Imperial College London, London, UK; 2 Faculty of Medicine, Airlangga University, Surabaya, Indonesia; 3 Institute for Human Development and Potential, Agency for Science, Technology and Research (A*STAR), Singapore; 4 Bioinformatics Institute, Agency for Science, Technology and Research (A*STAR), Singapore; 5 Strathclyde Institute of Pharmacy and Biomedical Sciences, University of Strathclyde, Glasgow, UK

**Keywords:** CORONARY ARTERY DISEASE, Global Burden of Disease, RISK FACTORS

## Abstract

**Background:**

Coronary artery disease (CAD) is one of the biggest causes of mortality worldwide. Risk stratification for early detection is essential for the primary prevention of CAD. QRISK3 is known to overestimate future CAD risk in some populations, resulting in unnecessary preventive treatment that reduces the cost-effectiveness and safety. Combining machine learning with a metaheuristic optimisation approach using the Particle Swarm Optimization algorithm may outperform QRISK3 in predicting CAD. It may improve performance by selecting the best-performing subset of features related to clinical outcomes.

**Methods:**

This study uses the UK Biobank dataset consisting of 348 015 participants aged 24–84 years with no prior diagnosis of CAD. The performance of both QRISK3 and machine learning models was evaluated separately using receiver operating characteristic analysis. Several machine learning models were assessed: Logistic Regression, Decision Tree, Random Forest, Naïve Bayes and Gradient Boosting. The dataset was split into training and test sets with a ratio of 4:1 for the machine learning models. Each model has been developed by adding a Particle Swarm Optimization algorithm to enhance the model’s classification accuracy.

**Results:**

Out of 348 015 participants, 23 136 individuals (6.64%) were diagnosed with CAD within 10 years following their first visit, while 324 879 individuals (93.4%) did not develop CAD. The area under the curve (AUC) value of the QRISK3 prediction was 0.6113, while the gradient boosting model using Particle Swarm Optimization achieved a better performance AUC of 0.7258.

**Conclusions:**

This study shows hybrid machine learning models optimised with the Particle Swarm Optimization algorithm can better predict CAD than QRISK3. The application of such machine learning models can effectively identify high-risk CAD patients, allowing for more personalised preventative strategies and supporting policymakers in implementing lifestyle change recommendations.

**WHAT IS ALREADY KNOWN ON THIS TOPIC**

- Conventional risk assessment tools such as Framingham Risk Score, pooled cohort equation, Systematic Coronary Risk Evaluation and QRISK3 have been widely used to predict an individual’s risk of developing coronary artery disease (CAD) and apply primary prevention.
- In previous studies, QRISK3 has been shown to overestimate the risk of CAD in most populations, reducing the cost-effectiveness of preventive treatment.
- Machine learning models potentially outperform QRISK3 in forecasting an individual’s risk of having CAD in the next decade.

**WHAT THIS STUDY ADDS**

- The combined machine learning models using the Particle Swarm Optimization algorithm, particularly the gradient boosting model, demonstrate superior performance compared with QRISK3 in predicting the probability of an individual acquiring CAD within a decade.
- The evaluation of QRISK accuracy using the UK Biobank indicated an area under the curve value of 0.6113, which remains insufficient for practical application in healthcare.

**HOW THIS STUDY MIGHT AFFECT RESEARCH, PRACTICE OR POLICY**

- The English National Health Service spent £250 million to bolster the use of machine learning in healthcare, underscoring the growing importance and potential of artificial intelligence in improving healthcare outcomes.
- At population level, risk prediction is critical in identifying individuals who would benefit from preventive treatment, primarily in identifying the need for a low-density lipoprotein cholesterol lowering agent, low-dose aspirin and blood pressure lowering agent. Therefore, an accurate risk prediction could minimise undertreatment and overtreatment, resulting in an improvement in safety and cost-effectiveness.
- These machine learning models can effectively identify high-risk CAD patients, allowing for more personalised preventative strategies and supporting policymakers in recognising population-level risks when implementing lifestyle change recommendations.

## Introduction

 Coronary artery disease (CAD) is a prevalent cardiovascular disease characterised by the build-up of atherosclerotic plaque inside the lumen of blood vessels.^1^ CAD accounts for approximately 33–50% of all cases of cardiovascular disease. It is a complex event influenced by several factors.^2 3^ While the progression of this disease may vary between countries, CAD remains a significant global health issue.^4^ The WHO reports that CAD results in approximately 17.9 million fatalities annually, representing 32% of global mortality. In 2015, the global prevalence of CAD was estimated at 8.9 million, but in 2022, it rose to 315 million. In the UK, CAD prevalence has remained stable at approximately 3% in England and 4% in Scotland, Wales and Northern Ireland. The number of hospital admissions for CAD rose by more than 46 000 between 2011 and 2014 and out of these increased admissions, more than 36 500 were for males.^5^ In the US, around 18.2 million individuals aged 20 years and older are affected by CAD, with a higher prevalence observed in men than in women.^6^ CAD also accounts for a significant portion of healthcare costs, reaching an estimated $126.2 billion in 2010.^5^ Nonetheless, there is a notable decrease in CAD across many nations attributable to the efficacy of primary prevention strategies. In 2022, the age-standardised prevalence of CAD was 3.605 per 100 000 globally, reflecting an 18% decrease from 1990, when it stood at 4.390 per 100 000.^7^

Precisely evaluating an individual’s risk for CAD is essential for effective primary prevention, facilitating early intervention aimed at modifiable risk factors. Numerous proven risk assessment instruments exist, such as the Framingham Risk Score, Systematic Coronary Risk Evaluation 2 (SCORE2), QRISK3, the Reynolds Risk Score and the ASCVD Risk Estimator.^8^

Of them, QRISK is extensively used in the UK. Launched in 2007 and subsequently revised, the most recent iteration, QRISK3, is available online. QRISK calculates the decade-long probability of developing cardiovascular conditions, including coronary heart disease, ischaemic heart disease and transient ischaemic attack. It assists healthcare providers in recognising high-risk individuals and executing suitable preventative measures.^9^

QRISK3 incorporated additional risk factors from the previous version, such as chronic kidney disease (CKD) stages 3–5, variability in systolic blood pressure (SBP), severe mental illness, migraine, routine corticosteroid administration, systemic lupus erythematosus, utilisation of atypical antipsychotic medications and the diagnosis or management of erectile dysfunction.^10^ The creation and verification of QRISK3 relied on comprehensive datasets from English general practices, consisting of a derivation cohort of 7.89 million patients (75%) and a validation cohort of 2.67 million patients (25%), aged 24–84 years.^10^ Notwithstanding its prevalent application, QRISK3 possesses limits that warrant consideration in clinical practice. Due to its development based on UK demographic data, its predicted accuracy may diminish when applied to groups with differing genetic, socioeconomic or demographic characteristics. A prior study indicated that merely 32% of QRISK3 predictions aligned with actual results, while receiver operating characteristic (ROC) analysis demonstrated an area under the curve (AUC) value of 0.60, signifying weak discriminatory efficacy.^11^ This could lead to unnecessary preventive treatment such as the use of statin therapy, which may reduce both cost-effectiveness and safety. In recent years, machine learning has been progressively used to improve risk prediction. Machine learning algorithms can evaluate extensive and intricate datasets, enhancing CAD detection, diagnosis and prognosis through increased speed, precision and personalisation.^12^ Previous studies indicated that machine learning models using electronic health records (EHRs) enhanced CAD risk assessment, presenting prospective advantages for preventive healthcare approaches.^13^

Nonetheless, constructing robust machine learning models is challenging, particularly with the problem of high dimensionality—the imbalance between the quantity of characteristics and the number of available samples. To overcome this, thorough evaluation of model generalisability and robustness is essential.^14^ Feature selection is an essential phase in the machine learning workflow that improves model efficacy and mitigates overfitting. The objective is to incorporate factors that are significantly correlated with the outcome while reducing irrelevant or redundant aspects.^14^ Feature selection can, in certain instances, be further refined by the application of metaheuristic algorithms that systematically investigate the solution space to enhance model accuracy. Ultimately, precise risk classification enhances individual clinical outcomes and aids policymakers in recognising essential public health goals and undertaking focused preventive treatment.^15^

## Methods

### Cohort dataset

This study presents a comparative analysis with a retrospective design to compare the performance between QRISK3 and hybrid machine learning models in predicting CAD. We use the data obtained from the UK Biobank; a population-based cohort established in 2006 in the UK that provides ten years of follow-up data from the initial assessment. The data were gathered from individuals between the ages of 24 years and 84 years, who registered between 2006 and 2010 without a prior diagnosis of CAD during the initial assessment. We further exclude individuals undergoing statin therapy, patients with missing follow-up data on CAD outcomes for 10 years post initial assessment and patients who died within 10 years of evaluation.

### Machine learning prediction of CAD

We assessed the performance of QRISK3 and machine learning models separately, mainly using ROC analysis, then compared the AUC value. The models include Logistic Regression, Decision Tree, Random Forest, Naïve Bayes and Gradient Boosting models. The numerical variables are standardised using Z-score normalisation. The data were randomly split into training sets and test sets in a 4:1 ratio and then proceeded to the learning phase. Within the machine learning models, we used a combination of a Particle Swarm Optimization (PSO) algorithm; a metaheuristic technique for selecting the most relevant features, which will allow us to identify the feature subsets that produced the highest-performing models in terms of accurately predicting CAD. The number of particles and iterations used for machine learning models were 15 and 80, respectively. Particles refer to the individual samples or solutions in a population-based algorithm, while iterations refer to the number of times the algorithm in the machine learning model updates its state or solution, contributing its convergence towards an optimal or satisfactory solution. In addition, accuracy, precision, recall and F1 score were calculated using a threshold of 0.5. For representation from both classes (positive and negative), the performance metrics were calculated using the macro average of precision, recall and F1-score. Python was used for all statistical analyses. The primary packages comprised numpy for numerical computations, pandas for data processing, scikit-learn for the development and evaluation of machine learning models and matplotlib and seaborn for data visualisation.

### QRISK3 prediction of CAD

20 variables—included in the QRISK3 assessment tool (https://qrisk.org/)—were selected as risk factors or feature variables. Due to unavailable data, we failed to use two variables from the QRISK3: (1) the SD of the two most recent SBP readings in mm Hg and (2) the UK Postcode.

The outcome of this study is to predict the occurrence of CAD within the next 10 years after the first assessment, which was described as coronary heart disease, ischaemic stroke or transient ischaemic attack. The QRISK3 model generates a numeric score that represents the probability of developing CAD. As per the criteria set by the National Institute for Health and Care Excellence (NICE) guidelines, risk levels are classified as low (below 10), moderate (between 10 and 20) and high (over 20). Subsequently, we categorised the predicted results into two distinct categories: low and moderate risk, which signifies the absence of CAD, and high risk, which indicates the presence of CAD. We used the International Classification of Diseases, 10th version clinical codes to ascertain instances from hospital and general practice data as the outcome.

## Results

From the original UK Biobank dataset including over 500 000 individuals, 348 015 participants aged 24–84 years without a prior history of CAD were selected for analysis. During a 10-year follow-up, 23 136 patients (6.64%) acquired CAD. CAD outcomes were defined using algorithmically-derived outcomes and the first occurrence fields, which combined information from multiple routine health data sources. These data were drawn from EHRs across England, Scotland and Wales. The data used for the machine learning models was split into a 4:1 ratio, resulting in a training set of 278 412 (80%) and a test set of 69 603 (20%). Out of the overall test set, 4626 individuals (0.07%) developed CAD. The study participants’ characteristics were as follows: 152 616 (43.9%) were male and 195 399 (56.1%) were female. The ethnic composition of the cohort is predominantly white or not stated, comprising 327 675 participants (94.2%). The data regarding first-degree relatives with a history of cardiovascular disease indicates that 216.097 (62.1%) do not have a familial history of the condition, whereas 131 918 (37.91%) had such a history. Regarding the comorbidities history, data show that 3602 individuals (1.04%) have atrial fibrillation (AF), 3189 (0.90%) have a history of CKD, 1.156 (0.30%) have diabetes mellitus (DM) type 1 and 6117 participants (1.80%) have DM type 2. None of the observed participants has a history of severe mental illness and rheumatoid arthritis. Out of the total participants, 28 786 (8.30%) are taking blood pressure lowering agent medication. The consumption of atypical antipsychotic medication is very rare, with just 803 individuals (0.23%) being prescribed such medication. Out of the population, 1917 individuals (0.55%) regularly consume oral steroid medication. Of the observed male participants, 868 (0.2%) of them consume erectile dysfunction medication. Table 1 provides a comprehensive description of the participants’ characteristics as observed during the initial assessment.

**Table 1:** Characteristics of UK Biobank participants included in the study

Participants’ characteristic	All participants (n=348.015)	Non-CAD outcome (n=324.879)	CAD outcome (n=23.136)	P value
Age	57 (95% CI: 41.0 to 69.0)	56 (95% CI: 55.97 to 56.02)	62 (95% CI: 61.91 to 62.08)	p<0.001
Sex				
Female	195 399 (56.1%)	186 176 (57.30%)	9223 (39.90%)	p<0.001
Male	152 616 (43.9%)	138 703 (42.70%)	13 913 (60.10%)	
Smoking status				
Non-smoker	143 641 (41.3%)	135 360 (41.70%)	8281 (35.80%)	p<0.001
Ex-smoker	105 283 (30.2%)	99 047 (30.50%)	6236 (27.00%)	
Light smoker	56 583 (16.3%)	52 415 (16.10%)	4168 (18.00%)	
Moderate smoker	32 230 (9.30%)	29 124 (9.00%)	3106 (13.40%)	
Heavy smoker	10 278 (2.90%)	8933 (2.80%)	1345 (5.80%)	
Chronic kidney disease				
No	344 826 (99.1%)	322 143 (99.20%)	22 683 (98.00%)	p<0.001
Yes	3189 (0.90%)	2736 (0.80%)	453 (2.00%)	
Type 1 diabetes mellitus				
No	346 859 (99.7%)	323 925 (99.70%)	22 934 (99.10%)	p<0.001
Yes	1156 (0.30%)	954 (0.30%)	202 (0.90%)	
Type 2 diabetes mellitus				
No	341 898 (98.2%)	319 810 (98.40%)	22 088 (95.40%)	p<0.001
Yes	6117 (1.80%)	5069 (1.60%)	1048 (4.60%)	
Ethnicity				
White or not stated	327 675 (94.2%)	305 878 (94.10%)	21 797 (94.20%)	p<0.001
Other ethnic group	6606 (1.90%)	6225 (1.90%)	381 (1.60%)	
Indian	4039 (1.16%)	3667 (1.10%)	372 (1.60%)	
Black Caribbean	3402 (0.98%)	3226 (1.00%)	176 (0.80%)	
Black African	2472 (0.71%)	2365 (0.70%)	107 (0.50%)	
Other Asian	1328 (0.38%)	1210 (0.40%)	118 (0.50%)	
Pakistani	1178 (0.34%)	1036 (0.30%)	142 (0.60%)	
Chinese	1167 (0.34%)	1141 (0.40%)	26 (0.10%)	
Bangladeshi	148 (0.04%)	131 (0.04%)	17 (0.07%)	
Atrial fibrillation				
No	344 413 (98.9%)	322 067 (99.10%)	22 346 (96.60%)	p<0.001
Yes	3602 (1.04%)	2812 (0.90%)	790 (3.40%)	
Severe mental illness				
No	348 015 (100%)	324 879 (100.00%)		p=1.00
Yes	0 (0.00%)		23 136 (100.00%)	
Rheumatoid arthritis				
No	348 015 (100%)	324 879 (100.00%)		p=1.00
Yes	0 (0.00%)		23 136 (100.00%)	
History of heart attack in first degree relatives				
No	216 097 (62.1%)	203 052 (60.20%)	13 045 (56.40%)	p<0.001
Yes	131 918 (37.9%)	121 827 (39.80%)	10 091 (43.60%)	
Blood pressure lowering agent medication				
No	319 229 (91.7%)	300 453 (93.60%)	18 776 (81.20%)	p<0.001
Yes	28 786 (8.30%)	24 426 (6.40%)	4360 (18.80%)	

**Figure 1:**
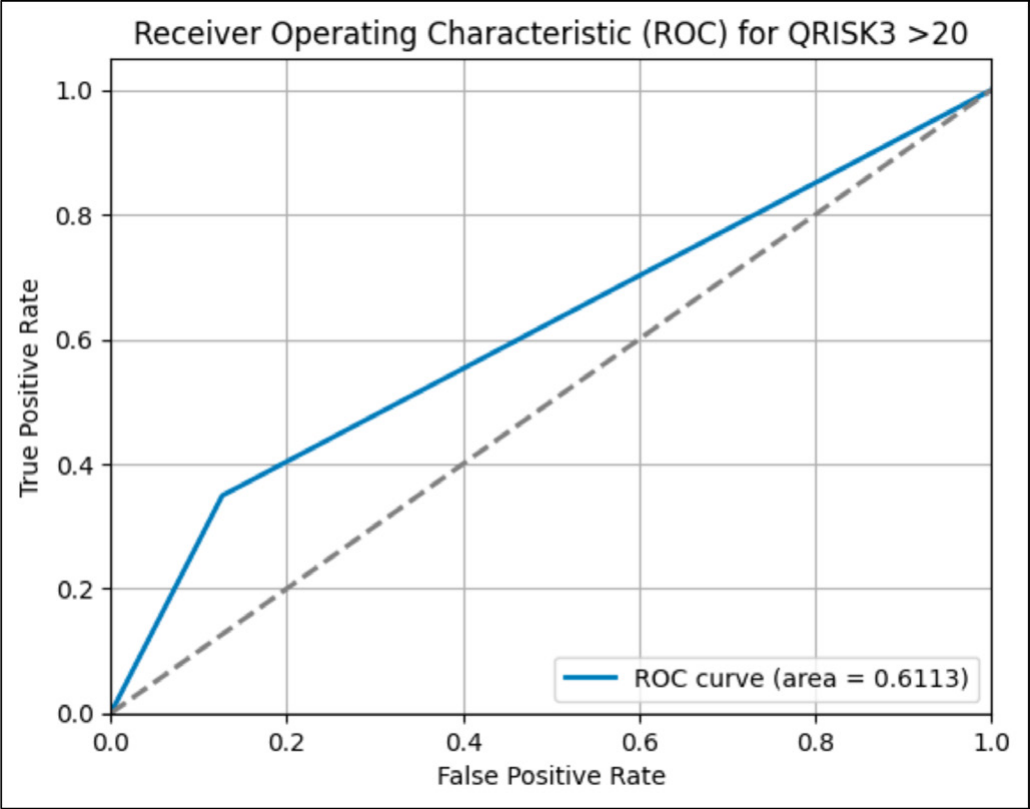
Receiver operating characteristic (ROC) analysis is being performed to evaluate the performance of QRISK3 in predicting future coronary artery dArtery Disease. The dashed line shows random classification performance, and the blue line is the ROC curve.

The prediction efficacy of the traditional QRISK3 tool was assessed in comparison to five machine learning models: Logistic Regression, Decision Tree, Random Forest, Naïve Bayes and Gradient Boosting, each further optimised by a PSO approach for feature selection. The QRISK3 model attained an AUC of 0.6113, as shown in figure 1, indicating poor discriminatory capacity, characterised by notably poor precision (0.16) and recall (0.35). Patients who are predicted to have positive CAD tend to be older and have higher body mass index (BMI), higher SBP and worse cholesterol ratios compared with those who are predicted to have a negative result without CAD. The CAD group exhibits a markedly greater proportion of males, a higher rate of smoking habits, chronic illnesses (CKD, type 2 DM, AF) and a familial predisposition to heart disease. In addition, they have a higher probability of being prescribed drugs for hypertension, psychiatric conditions, erectile dysfunction and corticosteroids taken orally. The distribution of ethnicities reveals a greater prevalence of individuals identifying as white.

**Figure 2:**
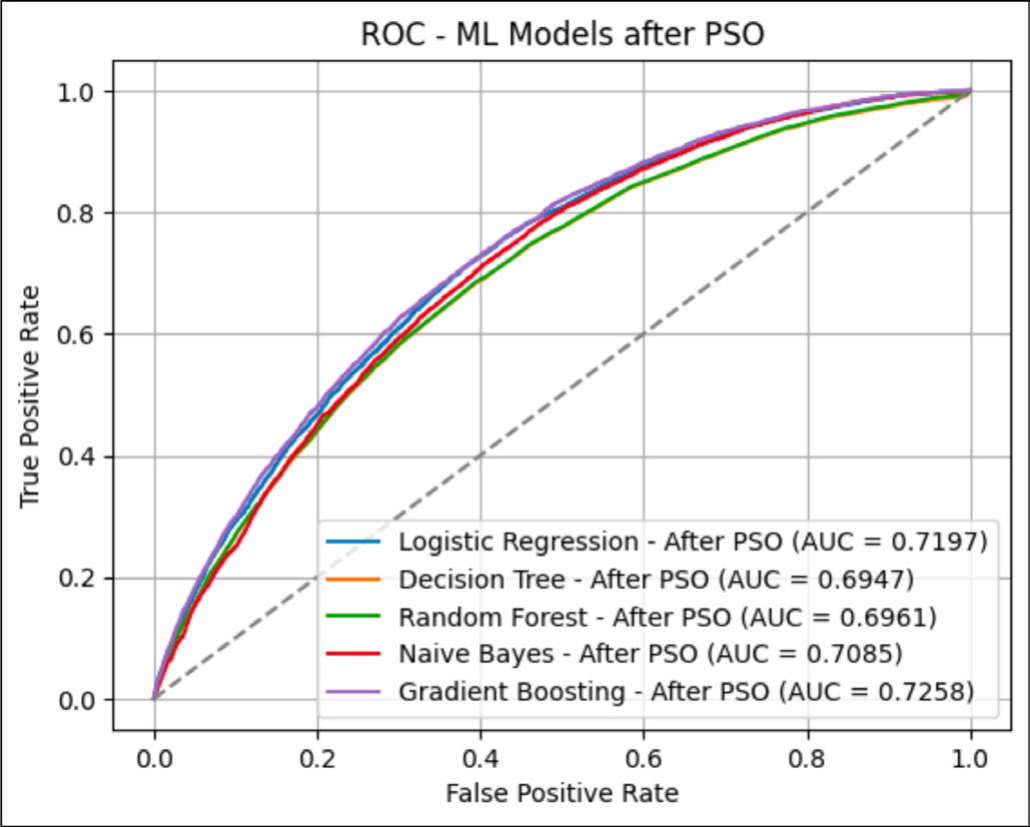
ROC analysis was performed on machine learning models after implementing the PSO algorithm. The dashed line shows random classification performance, and the coloured line is the ROC curve for each model. ML, machine learning; PSO, Particle Swarm Optimization; ROC, receiver operating charaOperating Characteristic.

**Figure 3:**
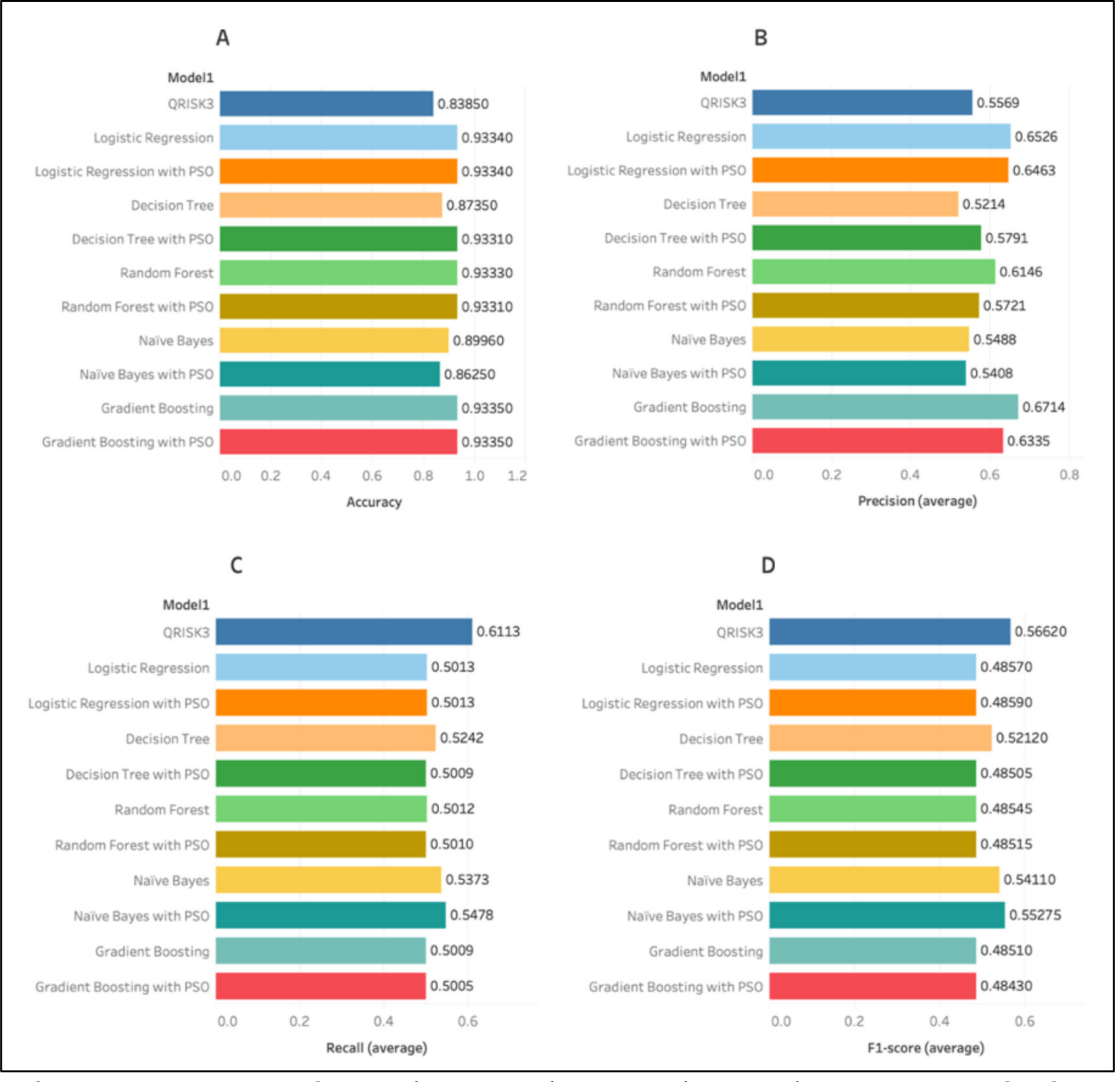
Performance comparison of various machine learning models and QRISK3 prediction. (**A**) Shows the accuracy, (**B**) shows the precision, (**C**) shows the recall and (**D**) shows the F-1 score. PSO, Particle Swarm Optimization.

The gradient boosting model with PSO exhibited superior performance compared with other machine learning methods, with an AUC of 0.7258 shown in figure 2 and surpassing QRISK3 in all assessed parameters, including precision (0.6714) and total accuracy (0.9335) shown in figure 3. While adding the PSO algorithm, we use multiple iterations using different subsets of features. The AUC values then range from 0.7253 to 0.7258. In the beginning, the curve demonstrates a swift rise in the AUC values over the first 10 iterations, suggesting a substantial enhancement in the model’s performance at the start of the optimisation process. Following the initial phase, the curve plateaus, indicating little improvements in the AUC values from the 10th to the 70th iterations. This suggests that the PSO algorithm has discovered a stable region where the model’s performance is only slightly enhanced. At approximately the 70th iteration, there is a subtle upward trend, culminating in a final increase in the AUC values, suggesting a minor improvement towards the conclusion of the iterations. At the 80th iteration, the curve hits its highest point, suggesting that the PSO algorithm has reached a nearly optimal solution for the gradient boosting model.

Although QRISK3 demonstrated marginally superior recall in detecting CAD cases, the machine learning models, especially Gradient Boosting and Naïve Bayes (AUC 0.7085), yielded a more equitable and precise classification overall. The implementation of the PSO method significantly improved AUC scores across all machine learning models, indicating its superiority towards improving feature selection and model performance for CAD risk prediction.

## Discussion

According to the result of the study using the UK Biobank cohort, the combined machine learning models with the PSO algorithm perform better than the conventional risk assessment, QRISK3, in predicting the risk of developing CAD within a 10-year period. The combination of the Gradient Boosting model with the PSO algorithm achieved the highest AUC values (AUC=0.7258) in classifying outcomes and is the best-performing model. This indicates that the model is appropriate and capable of distinguishing between positive and negative predictions.^16^ The AUC value of Gradient Boosting with the PSO algorithm was +0.1143 higher than that of the conventional pre-existing risk prediction tool, QRISK3. The findings of this study align with prior studies that have examined the comparison between machine learning models and conventional risk assessment tools, such as the Pooled Cohort Equations (PCEs), where the machine learning model demonstrated significantly superior performance.^17^ Machine learning offers a new approach to risk stratification, potentially resulting in more accurate and specific predictions compared with classic regression-based statistical models.^18^

The Gradient Boosting model is built on the fundamental notion of boosting, which is a highly important strategy in the field of machine learning. The approach involves iteratively acquiring a collection of multiple weak learners to build a more advanced learning model that achieves superior performance. It uses the gradient descent technique to select a weak learner function in each step based on the negative gradient of the loss function. The primary objective of boosting is to identify the most effective function of variables for predicting the outcome while simultaneously minimising the loss function.^19^

The implementation of the PSO algorithm resulted in a slight increase in the AUC value of the models. It proved to be a valuable approach for identifying the most relevant subset of features from the QRISK3 dataset, hence facilitating the learning process of the models. Nevertheless, the AUC value in the Gradient Boosting model only improves by +0.0005 after applying the PSO algorithm, indicating minimal optimisation. Meanwhile, the decision tree model shows a more significant optimisation of +0.1705. The PSO algorithm is combined with Gradient Boosting analysis, incorporating various relevant features such as age, sex, smoking status, CKD, type 2 DM, ethnicity, AF, severe mental illness, history of angina or heart attack in the first-degree relatives, SBP, BMI, cholesterol/high-density lipoprotein (HDL) ratio, consumption of blood pressure lowering agent medication, atypical antipsychotic medicines, erectile dysfunction medication and oral steroid medication. This finding aligns with previous research that demonstrated the effectiveness of employing the PSO algorithm as a metaheuristic optimisation strategy for feature selection, leading to a significantly enhanced model performance.^20^,^23^ As pointed out in a prior study, the results of the PSO algorithm may vary based on the classification models employed.^24^ Since the dataset from the UK Biobank only includes similar features with QRISK3, not a very high number of variables compared with the data itself, the PSO algorithm in this case is less influential than expected. If we include all the possible available features (the ones that are relevant and not), the PSO algorithm might optimise the model more significantly.

### Importance of improving risk prediction models in healthcare

A comprehensive risk assessment is crucial for healthcare prevention at the individual and population levels.^25^ Several countries have established their own set of tools for evaluating potential risks: Americans use the PCE, Europeans use the SCORE2 and the UK employs QRISK3. At the individual level, assessing CAD risk enables individuals to become aware of the importance of managing risk factors for the prevention of CAD. Thus, specific efforts could be made for prevention strategies. By assessing a person’s risk, healthcare professionals can set exact targets and monitor adherence to lifestyle and behavioural recommendations to each patient.

At the population level, risk prediction is critical in identifying individuals who would benefit from preventive treatment to improve the cost-effectiveness and safety of the community. These treatments include pharmacological therapies such as low-density lipoprotein cholesterol lowering agent, low-dose aspirin/anti-platelet therapies, blood pressure lowering agent, high-blood sugar lowering agent and anti-thrombotic therapy.^26^ Prior research indicates that early statin therapy could offer advantages and is markedly linked to a reduced risk of CAD.^27 28^ Nonetheless, statins remain underprescribed, partly due to concerns about their possible adverse effects.^28^ This approach aims to prevent both undertreatment and overtreatment in older individuals.^25^ To achieve this goal, it is critical to minimise both overestimation and underestimation when predicting the risk of developing CAD.

Our model functions as a binary classifier, yielding two potential outputs: ‘Yes – the patient has CAD’ or ‘No – the patient does not have CAD’. The main objective was to assess the precision of this binary method, as clinical decision-making frequently results in a tangible differentiation between the presence or absence of disease. Although this method may pose a risk of underdiagnosis, the model was intentionally tuned for increased specificity, hence minimising inappropriate prescriptions of preventative drugs like statins and improving cost-effectiveness in public health systems.

This idea supports governments and healthcare providers in resource allocation by enabling them to distinguish between patients who may be prioritised for further evaluation and require more invasive treatment and those who can still focus on disease prevention through lifestyle modifications.

### The QRISK3 performance

The AUC value obtained for QRISK3 is 0.61 and is still deemed inadequate in the context of healthcare and its practical use. Typically, an AUC value of 0.80 or higher is considered as good.^16^ The study found that QRISK3 predicted that 49.209 (14.14%) of the overall population would develop future CAD, with an accuracy of 0.8385. However, the actual outcome showed that only 23 136 (6.64%) individuals developed CAD. Previous research has demonstrated that QRISK3, when applied to the Korean and Chinese populations, also tends to overestimate the risk compared with the actual occurrence of CAD. This discrepancy may be attributed to variations in ethnic backgrounds or the prevalence of CAD in Asian countries.^17 25 29^ Nevertheless, the study of a European population exhibited a significant overestimation of the actual result, indicating the presence of other influences beyond just ethnic origin. The tendency to overestimate may result in excessive treatment of risk factors, such as the use of statin medication for hypercholesterolaemia.^17 25 29^

### Gradient Boosting model with PSO algorithm approach

The data separation for the training set and the test set followed a 4:1 ratio, with 278 412 participants allocated to the training set and 69 603 participants allocated to the test set. The test set indicates that the actual outcome for CAD is 4.626, accounting for 0.07% of the entire test set. The model demonstrates a significant underestimation of the proportion of females in the CAD group, with a predicted value of 6.67% compared with the actual value of 40.51%. Conversely, the model overestimates the proportion of men, with a predicted value of 93.33% compared with the actual value of 59.49%. The model’s predictions are very similar to the actual classification in terms of smoking status. The model’s predictions for the proportion of individuals without CKD in the CAD group (73.33% predicted vs 97.80% real) are lower than the observed values, suggesting that the existence of CKD is less frequent than what is actually observed in the CAD group. The model underestimates the percentage of individuals without type 2 DM in the CAD group. The projected proportion is 46.67%, while the real proportion is 95.59%. The model’s predictions for the ‘white or not stated’ category exhibit a high level of preciseness when compared with the actual data. The model underestimates the percentage of persons without AF in the CAD group. The projected proportion is 40.00%, while the real proportion is 96.45%. The model underestimates the percentage of persons in the CAD category who are not taking blood pressure-lowering medications (13.33% predicted vs 81.17% actual).

Previous study has shown that machine learning models that ignored censoring, substantially underestimated risk of cardiovascular disease. A patient with high risk in QRISK3, with a score of 9.5%–10.5%, had a risk of 2.9%–9.2% in a random forest and 2.4%–7.2% in a neural network.^30^ The optimum results from machine learning models also need to be considered as there is a wide variety between and within different types of machine learning models resulting in the consistency of variety models. Consequently, different treatment decisions could be made by arbitrarily selecting another modelling technique.^30^

### Limitations and bias

A primary limitation of this study is the quality and comprehensiveness of the input data and features, which directly affect the learning process of the machine learning models. First, the data source used in this study does not provide information on the poverty status or level of deprivation associated with UK postcodes, which might offer a significant socioeconomic perspective.^31^ There is also an absence of data regarding SBP variation. The dataset contains individuals with CKD, but it does not define the stage of the illness. This is despite the initial goal to only include individuals with stage 3, 4 and 5 CKD based on the QRISK3 predictions. Reporting angina or heart attacks in first-degree relatives before 60 is crucial, but existing data only show their presence without defining the age of occurrence, which may limit results.

Another limitation is that the comparison between the two tools, the machine learning models and QRISK3, was that the dataset used for the machine learning models was split into two parts with a 1:4 proportion (test set and training set). Therefore, not 100% of the data were analysed in the test set, resulting in less data being used for testing, which might have affected the results.

The study did not include cross-validation processes, which could have provided a more reliable assessment of model performance and mitigated the risk of overfitting or bias resulting from a singular data split.

### Future direction

The optimisation of machine learning development needs a careful balance between the cost and achieving peak performance in healthcare settings, especially considering the UK National Health Service’s significant investments.

Further research is required to include a more extensive sample of people with varied sociodemographic traits to enhance the model’s adaptability to numerous scenarios. Additionally, an independent population study should be conducted to provide a benchmark, ensuring that the model’s performance is tested against varied and representative cohorts.

It is essential to evaluate the QRISK results between the initial assessment and the final outcome about the incidence of CAD. Could the low AUC be attributable to the patients’ initial awareness of their increased risk for CAD, which prompted them to change their lifestyle and adopt preventative measures, thereby delaying the development of the disease? It is important to evaluate the idea’s impact in real-world clinical settings.

## Conclusion

This study demonstrates that combined machine learning models optimised with the PSO algorithm showed a modest improvement in predicting CAD compared with the QRISK3. Allocating healthcare resources, ensuring high-risk patients receive preventative therapies and decreasing wasteful treatments due to overestimation require accurate risk prediction systems. This method helps policymakers identify population-level dangers and promote lifestyle changes.

## Data Availability

Data are available in a public, open access repository.
